# Does variability in recognition memory scale with mean memory strength or encoding variability in the UVSD model?

**DOI:** 10.1177/17470218221136498

**Published:** 2022-11-21

**Authors:** Rory W Spanton, Christopher J Berry

**Affiliations:** School of Psychology, Faculty of Health, University of Plymouth, Plymouth, UK

**Keywords:** Recognition memory, memory strength, encoding variability, strength scaling, unequal variance

## Abstract

The unequal variance signal detection (UVSD) model of recognition memory assumes that the variance of old item memory strength (σ_o_) is typically greater than that of new items. It has been suggested that this *old item variance effect* can be explained by the encoding variability hypothesis. However, Spanton and Berry (2020) failed to find evidence for this account, suggesting that σ_o_ may simply scale with mean memory strength (*d*) in the UVSD model. Experiments 1 and 2 examined the effects of encoding variability and strength scaling on old item variance by creating conditions in which mean memory strength and variability in item characteristics was either low or high in 2 × 2 factorial designs. In Experiment 1, overall strength determined estimates of σ_o_, with no effect of item characteristic variability. The same effect of overall strength was found in Experiment 2; there was also a significant effect of item characteristic variability, although this manipulation also had some effect on *d* and was therefore partially confounded. Experiment 3 similarly found a simultaneous increase in old item variance and memory strength in a design using mixed item characteristic variability conditions in a single-study/test block. We conclude that old item variance increases with mean memory strength in the UVSD model, with uncertainty about the effects of encoding variability, and that future explanations of the old item variance effect should bear this in mind.

In a recognition memory test, participants judge whether they have previously seen items in a particular context. Inevitably, some of these items are remembered better than others. This can be represented in a signal detection model wherein items at test are associated with a “memory strength” (henceforth “strength”) variable. The strength of “old” items (those which have been seen in a study phase) and unstudied “new” items are represented as separate Gaussian distributions along a unidimensional continuum. Because of exposure at study, the mean of the old item distribution is generally greater than that of the new item distribution, reflecting a difference in overall strength between the two item types. The difference between these means (*d*) is therefore a measure of recognition performance. Recognition memory judgements are modelled by comparing the strength value of a given item to static criteria along the strength continuum that correspond to different levels of confidence that an item is either old or new. These may range from high confidence that an item is new nearer to the lower end of the continuum to high confidence that an item is old towards the higher end of the continuum.

Although both new and old items vary in strength, it is widely accepted that the variance of the old item strength distribution (σ_o_) is greater than the variance of the new item distribution (see Rotello, 2017, for a review). The acceptance of this *old item variance effect* is motivated by analyses of the *z*-ROC, a *z*-transformed plot of the probability of correctly judging an old item “old” against the probability that a new item is incorrectly judged “old” at each level of recognition confidence in a given response scale. It is commonly found that *z*-ROCs calculated from recognition confidence data are approximately linear, with slopes less than 1 (Glanzer et al., 1999). Since the value of the *z*-ROC slope has long been presumed to represent the ratio σ_o_/σ_n_ in a traditional Gaussian signal detection model (but see Rabe et al., 2021), a non-unit *z*-ROC slope necessitates making σ_o_ a free parameter with a value typically greater than σ_n_. With this parameterization, the unequal variance signal detection (UVSD) model is defined as having parameters θ = {*d*, σ_o_, *C*_1_, *C*_2_, . . . *C_I_*} where *I* is the highest decision criterion level in terms of strength (Kellen et al., 2013). Therefore, the probability of a “hit” response (a correct “old” judgement) at criterion *i* according to the model is

$$P(H)=\Phi \left(\frac{d-C_{i}}{\sigma_{o}}\right)$$

where Φ is the cumulative normal distribution function. The probability of a “false alarm” response (incorrectly judging a new item “old”) at *C_i_* is

$$P(FA)=\Phi (-C_{i})$$

Although the UVSD model can account for some commonly observed regularities in the *z*-ROC slope (Egan, 1958; Yonelinas & Parks, 2007), its unequal variance assumption was created purely for the need to account for observed data, and not with a priori psychological assumptions in mind. However, a complementary psychological explanation for the unequal variance assumption was later proposed in the form of the encoding variability hypothesis (Jang et al., 2012; Wixted, 2007). According to this theory, the old item variance effect is caused by the presence of a large number of variables that affect memory strength at encoding. These variables contribute additional strength and variance to memory strength across a set of old items during the study phase, resulting in an increase in σ_o_ relative to σ_n_. Examples of such *encoding variables* could presumably include the level of attention paid to a stimulus, item characteristics, item-participant interaction, and many others. Stated mathematically, old items have some level of baseline strength, *B ~ N*(µ_baseline_, σ_baseline_), which is equivalent to the new item strength distribution (Jang et al., 2012). In the study phase, *B* is incremented by an added strength variable *A ~ N*(µ_added_, σ_added_) during encoding. The addition of baseline and added strength gives the resulting old item distribution in the formula *O* *=* *B* *+* *A*.

There have been several attempts to test the encoding variability hypothesis and compare its predictions with those of other accounts. Koen and Yonelinas (2010) first attempted this in a method where items at study were presented for either a fixed duration of 2500 ms, or a mixture of 1000 and 4000 ms durations. It was found that the latter variable encoding condition did not change estimates of σ_o_. Instead, the contribution of an additional recollection process was solely responsible for changes to the *z-*ROC slope, supposedly constituting evidence against the encoding variability hypothesis in favour of a dual-process model. However, subsequent comments by Starns et al. (2012) and Jang et al. (2012) clarified that these results had no bearing on the encoding variability hypothesis. This was because Koen and Yonelinas’s (2010) method mixed two discrete levels of encoding strength, which would be expected to result in a mixture strength distribution rather than a Gaussian as the encoding variability hypothesis predicts. However, Koen et al. (2013) later studied the effects of retrieval manipulations on old item variance, finding that it was possible to induce changes in estimates of σ_o_ without manipulating encoding variability. Although this finding does not exclude the possibility that encoding variability may still have some role in determining estimates of σ_o_, it suggests that it is not the only factor that influences old item variance.

More recently, Spanton and Berry (2020) attempted to test the encoding variability hypothesis by manipulating encoding variables directly during study. To avoid the creation of mixture strength distributions that confounded Koen and Yonelinas (2010), encoding variables were manipulated by adding variance along a continuous scale, rather than by mixing two separate conditions of high or low quality encoding. Across three experiments, attempts to influence σ_o_ by manipulating three encoding variables (study duration, attention, and word frequency) were unsuccessful; there were no resultant effects on σ_o_, although each manipulation was assessed to have a weak effect on recognition confidence ratings. Despite this, both *d* and σ_o_ were found to be significantly greater in the low item characteristic variance condition in Experiment 2, suggesting again that changes in σ_o_ may result from factors other than encoding variability. Estimates of *d* and σ_o_ also showed strong positive correlations in every experiment, indicating that old item variance may scale with mean strength. This was not predicted by the encoding variability hypothesis.

The idea that mean memory strength and variance in memory strength are related is evidenced elsewhere in the recognition memory literature. Although some previous research concluded that the *z*-ROC slope takes a constant value of approximately 0.8 (Ratcliff et al., 1994; Ratcliff et al., 1992), it was later found that in many cases, increases in mean strength generally decrease the *z*-ROC slope (Glanzer et al., 1999; Parks & Yonelinas, 2007), meaning that mean strength and old item variance increase with one another in several experimental contexts. The finding that greater strength coincides with greater old item variance has since been observed in other studies (Glanzer & Adams, 1990; Heathcote, 2003; Hirshman & Hostetter, 2000; Koen et al., 2013; but see Grider and Malmberg, 2008; Starns et al., 2012). More recently, Dopkins et al. (2017) found that a semantic priming manipulation increased the memory strength of new items and the variance of their corresponding confidence ratings at test, as well as the *z*-ROC slope. This suggests that a form of strength and item variance scaling could apply more generally to both old and new item types—a distribution with a greater mean tends to have a greater variance. In sum, this is evidence that σ_o_ scales as a monotonically increasing function of *d* in many experimental settings.

Our first two experiments aim to test whether estimates of σ_o_ are affected by encoding variability or mean memory strength. To achieve this, a successful manipulation of encoding variability during the study phase is needed. Despite previous efforts by Spanton and Berry (2020) to add Gaussian variability to individual item characteristics, the resultant effects upon old item variance were weak. This may be because even without experimental manipulation, there are already a very large number of encoding variables that sum to determine levels of added strength in any condition. Therefore, any further attempts to experimentally manipulate a given encoding variable might have a minimal effect on old item variance because added strength already varies greatly. It could also be possible that the effect of any experimentally manipulated encoding variable is partially counteracted by any number of other encoding variables that occur naturally. When manipulating item characteristics, for example, if word frequency and strength are negatively related, whereas concreteness and strength are positively related, then any amount of added strength that a word may receive for having low word frequency may be balanced by a decrement in strength if that word also happens to have low concreteness. Furthermore, there is likely to be a negative correlation between an item’s baseline strength value and the increment of added strength it receives during study (Jang et al., 2012), which, in conjunction with the aforementioned factors, could make it difficult to establish a strong experimental manipulation of encoding variability (Spanton & Berry, 2020).

A potential way to address these problems is to manipulate multiple item characteristics simultaneously to achieve a greater combined experimental effect upon old item variance. In doing so, the possibility that manipulated item characteristics may systematically counteract each other can also be addressed by ensuring that these characteristics are correlated within a word list. Returning to the example above, word frequency and concreteness would be less likely to counteract one another if their values were negatively correlated, increasing their summated effect upon the variance of recognition confidence judgements. Such a condition could be compared with another wherein item characteristics are constrained to be as low in variance as possible, resulting in low encoding variability. Furthermore, if the mean of each item characteristic measure is equal across word lists in both high and low variability conditions, the overall memorability of stimuli in each set would be controlled. This control of overall stimulus memorability within an item characteristic manipulation allows for memory strength to be manipulated orthogonally as a separate factor.

Our third experiment attempts to test the encoding variability hypothesis by including low and high item characteristic variance stimulus conditions within a single test phase, rather than separate ones. In each condition of Experiments 1 and 2, the characteristics of old and new items had approximately equal variance. This prevents some words in high encoding variability lists being artefactually more discriminable based on their extreme characteristics, which would confound the orthogonal manipulation of mean memory strength. However, as σ_o_ is conceptualised as the ratio of new/old item variance in the UVSD model, it is possible that our item characteristic manipulations would not affect this parameter, unless old items gain added variability purely by virtue of being studied (Wixted, 2007). Experiment 3 addresses this possibility to provide a new test of the encoding variability hypothesis. Experiments 1 and 2 were preregistered on the Open Science Framework (https://osf.io/ty8vz/), with details of our main hypotheses, experimental designs, methods, and analyses being disclosed before data collection for each respective experiment. Deviations from our preregistration were also disclosed. Materials, data, and analyses from Experiment 3 are also found in our OSF repository.

## Experiment 1

In the following experiment, we manipulate both variability in item characteristics and memory strength at two levels each (high, low) in a 2 × 2 factorial design. Strength will be manipulated using a one-back digit judgement task identical to that in the “fixed” condition in Experiment 2 of Spanton and Berry (2020). This task will be present as a simultaneous distraction in low strength condition study phases and absent in high strength conditions. In high variability conditions, words will be selected in a manner that attempts to ensure that they have high Gaussian variance in terms of four normalised variables previously shown to influence memory strength: (1) word frequency, which was shown to have significant effects on various recognition memory accuracy metrics in multiple studies (Glanzer & Bowles, 1976); (2) concreteness, shown to have a roughly 8% effect on correct recognition rate by Fliessbach et al. (2006); (3) age of acquisition (AOA; Cortese et al., 2010), shown to have a weak-moderate association with recognition confidence ratings; and (4) word length, which was shown to have a moderate negative relationship with correct recognition rate (Cortese et al., 2010, 2015).

Besides word length, each variable will be inter-correlated to promote maximal effects upon recognition confidence ratings. In contrast, words in low variability conditions will have low variance in terms of the above variables (and a fixed word length), with mean word frequency, concreteness, and AOA scores equal to those in high variability conditions. After fitting the UVSD model to the data, we expect a main effect of our strength manipulation on *d*, with no main effect of item characteristic variability on *d*, and no interaction. Given this outcome, if mean memory strength influences old item variance, we expect a main effect of strength on σ_o_, with no main effect of item characteristic variability and no interaction. In contrast, if the encoding variability hypothesis holds (and is represented by our manipulation of item characteristics), we would expect a main effect of item characteristic variability on σ_o_ with no main effect of strength and no interaction.

### Methods

#### Participants

In all, 64 participants (12 males, 52 females) with a mean age of 22.30 (*SD* = 8.78) from the University of Plymouth Psychology Participation Pool took part in this experiment. Each participant was a University of Plymouth psychology undergraduate, fluent in English as a first language and not dyslexic. Participants received course credits or £8 cash payment for their participation. We justified our sample size on the basis that it was compatible with our partial counterbalancing design (see Design and Procedure), and that it gave us sufficient power to detect a small-medium effect size, that is, Cohen’s *f*(*V*) = .36, with α = .05 and .80 power in a 2 × 2 within-subjects ANOVA. This experiment, along with the others in this article, was conducted with ethical approval from the University of Plymouth Faculty of Health Ethics Committee.

#### Materials

A total of 480 unique words were used as stimuli (60 old and 60 new in each condition). Chosen words appeared in the SUBTLEX-UK word database (Van Heuven et al., 2014) and databases from Brysbaert et al. (2014) and Kuperman et al. (2012). Names, proper nouns, and hyphenated words were excluded from an aggregate of the above databases before sampling. Word frequency scores for these words were taken from the SUBTLEX-UK database (Van Heuven et al., 2014), concreteness scores were taken from Brysbaert et al. (2014), and AOA scores were taken from Kuperman et al. (2012). In high item characteristic variability conditions, each set of old or new words (four in total) was selected using an algorithm with the following criteria:

1. Words must be 4–10 characters long.
2. Each set of words must have approximately equal mean word frequency (~3), concreteness (~3), and AOA (~10) scores (see Table 1 for exact values).
3. Concreteness and AOA scores must be strongly negatively correlated with word frequency scores within each word list (*r* < −.77 for concreteness and word frequency scores, *r* < −.61 for AOA and word frequency scores, and *r* > .26 for concreteness and AOA scores).
4. The distribution of word frequency, concreteness and AOA scores must not significantly deviate from a normal within each set, according to an Anderson-Darling test (*p* > .05).

**Table 1.** Mean word frequency, concreteness, and age of acquisition scores in Experiments 1 and 2, with standard deviations in brackets.

Encoding variable	Word list	Experiment 1		Experiment 2	
		High variability	Low variability	High variability	Low variability
Word Frequency	1	2.94 (0.94)	2.95 (0.31)	2.94 (1.21)	2.99 (0.38)
	2	2.88 (0.92)	2.92 (0.36)	2.95 (1.26)	2.97 (0.37)
	3	2.87 (0.95)	2.97 (0.33)	2.89 (1.22)	2.94 (0.34)
	4	2.89 (0.93)	2.94 (0.32)	2.95 (1.26)	2.90 (0.37)
Concreteness	1	3.10 (0.87)	3.14 (0.44)	3.10 (1.14)	3.07 (0.36)
	2	3.09 (0.87)	3.07 (0.51)	3.12 (1.08)	3.18 (0.49)
	3	3.09 (0.87)	3.17 (0.51)	3.11 (1.21)	3.11 (0.48)
	4	3.05 (0.82)	3.09 (0.41)	3.07 (1.12)	3.14 (0.47)
Age of Acquisition	1	10.30 (2.42)	10.40 (0.56)	10.40 (3.33)	10.30 (0.53)
	2	10.30 (2.31)	10.40 (0.51)	10.40 (2.98)	10.30 (0.49)
	3	10.30 (2.52)	10.40 (0.48)	10.40 (3.40)	10.40 (0.58)
	4	10.40 (2.25)	10.20 (0.56)	10.20 (3.69)	10.30 (0.44)

The remaining four sets of old/new words in the low item characteristic variability condition were sampled with the following criteria:

1. Words must be seven characters long.
2. Each set of words must have approximately equal mean word frequency, concreteness, and AOA scores (with the same constraints as the high item characteristic variance condition).
3. Each item characteristic variable must not be highly correlated. Among the word lists generated, word frequency and concreteness had a maximum negative correlation of *r* = −.36. Word frequency and AOA had a maximum negative correlation of *r* = −.11. Concreteness and AOA had a maximum positive correlation of *r* = .03.
4. Word frequency, concreteness and AOA scores must have low variance. For each word, the formula Σ|(μ*_e_—e_i_*)| was used to determine the summed difference between the mean of each item characteristic (*e*) across all possible words, and its corresponding value in the *i*th word. The 240 words with the lowest summed difference scores were then randomly sampled from without replacement to create the low encoding variability word lists.

In low strength conditions, participants heard audio clips of a female computer-generated voice speaking a number between 1 and 9 in each trial; this audio was absent in high strength conditions. The whole experiment was conducted on Lenovo desktop computers running an OpenSesame programme (Mathôt et al., 2012) which displayed all stimuli, instructions, and logged response data. Stimuli were presented in 40 pt “mono” font.

#### Procedure

Participants completed all four experimental conditions in a within-subjects design. The order of conditions, the order of high item characteristic variability word lists, and the order of low item characteristic variability word lists were all partially counterbalanced according to a Latin square. This resulted in a 4 × 4 × 4 partial counterbalancing design. All participants gave informed consent using a keypress response.

Before participants began their first low strength condition, they completed practice trials where they responded to auditory distractor digits without having to remember items simultaneously. In these practice trials, a fixation point was presented for 500 ms, followed by an auditory digit and a simultaneous visual prompt to respond to the digit from the previous trial. This prompt appeared in the centre of the screen, lasting 3000 ms (on the first practice trial, participants were prompted to make no response as there was no previous trial). This was followed by a 500 ms inter-trial interval (ITI), during which no information was presented in the centre of the screen. The key “*Z* = Previous number even, M = Previous number odd” remained static near the bottom of the screen for the duration of the practice trials; participants made responses when prompted using the Z and M keys as instructed. To advance to the following study phase, participants had to make eight consecutive correct responses; if they did not do so after 30 trials, the experimenter would re-explain the task to the participant before they attempted the practice trials again.

In each condition of the main experiment, participants then completed a 60-trial study phase. The low strength study phases shared the same trial level procedure as the practice phase, with the exception that instead of a prompt to respond to the previous number, a randomly selected old word was presented in the centre of the screen. In high-strength conditions, participants did not have to complete a simultaneous one-back task. Features associated with this task were therefore not present in these conditions, such as the auditory digits and the response key, although the duration of the fixation, stimulus presentation and ITI remained the same. In all conditions, participants were instructed to try their best to pay consistent attention to each word during study.

In between study and test phases in each condition, participants completed a short retention interval in which they answered basic arithmetic questions. These questions took the form “*A* ± *B* ± *C* = ?” where *A*, *B*, and *C* were one or two digit positive integers. The correct answer was always a one or two digit integer. Participants completed sequential trials of these questions for 60 s, at which point they progressed to the test phase.

In every condition, test phases were identically structured; a fixation point would appear for 500 ms, followed by a randomly selected word that was either old or new in the centre of the screen. This word was presented until the participant made a recognition confidence judgement based on their degree of certainty that the item was old or new. Participants made these responses with 1–6 keys at the top of the keyboard, using the category scale “1—Sure New, 2—Probably New, 3—Guess New, 4—Guess Old, 5—Probably Old, 6—Sure Old.” This key, and the prompt “New or Old?” were presented near the bottom of the screen as a static reminder of the response categories throughout each test phase. After each response, a 500 ms ITI (in which no information was displayed in the centre of the screen) was displayed, before the next trial. Participants were instructed to make use of the whole rating scale, and to prioritise the accuracy of their judgements over speed as they completed the task.

### Results

All analyses were conducted in the statistical programming language *R* (Version 4.2.0; R Core Team, 2022), primarily using the *tidyverse* package (Wickham et al., 2019). All Bayes Factors (Scaled JZS) were reported using the *BayesFactor* package (Morey & Rouder, 2018). The UVSD model was fit to the data using maximum likelihood estimation (Dunn, 2010).

In the following analyses, we excluded four participants who predominantly used the “Sure New” and “Sure Old” responses, resulting in large outlying parameter estimates (over 3 standard deviations above the mean estimates for σ_o_ and *d*). We did so because these data did not give a meaningful representation of variability in recognition responses, and because we defined this criterion for exclusion in our preregistration. We also analysed the natural logarithmic transformation of σ_o_ because, with the value of σ_n_ fixed to equal 1, σ_o_ is a ratio and would otherwise violate the assumptions of a 2 × 2 ANOVA.

#### Study task performance

The proportions of correct responses made in each “low strength” study phase condition were compared to check whether the presence of an item characteristic variability manipulation resulted in any task interference effects. The mean proportion of correct one-back task responses did not differ significantly between the “Low Strength, High Variability” condition (*M* = .94, *SE* = .01) and “Low Strength, Low Variability” condition (*M* = .94, *SE* = .01), *t*(59) = 0.34, *p* = .74, 95% CI: [−0.02, 0.02], BF = 0.15.

#### Item characteristic variability manipulation

To confirm whether our manipulation of item characteristic variability influenced subsequent recognition ratings, multiple regression analyses were conducted within each condition for each participant. Word Frequency, Concreteness, Age of Acquisition and Word Length were specified as predictors of recognition confidence ratings for each old item at test. The proportion of significant regression models (as assessed by the *F-*statistic) and mean *R*^2^ values for each condition are reported in Table 2.

**Table 2.** The proportion of significant regression models and mean *R*^2^ values (standard deviations in brackets) for each condition in Experiments 1, 2, and 3.

Experiment	Condition	*P*(significant) regressions	Mean *R*^2^
Experiment 1	High Strength, High Variability	.22	.11 (.07)
	High Strength, Low Variability	.10	.06 (.04)
	Low Strength, High Variability	.23	.12 (.07)
	Low Strength, Low Variability	.03	.06 (.04)
Experiment 2	High Strength, High Variability	.38	.13 (.08)
	High Strength, Low Variability	.07	.05 (.04)
	Low Strength, High Variability	.22	.12 (.09)
	Low Strength, Low Variability	.05	.05 (.04)
Experiment 3	Old, High Variability	.19	.09 (.06)
	Old, Low Variability	.04	.05 (.04)
	New, High Variability	.19	.08 (.07)
	New, Low Variability	.04	.06 (.04)

To compare these *R*^2^ values, we conducted a 2 × 2 ANOVA on *R*^2^ with strength (high, low) and item characteristic variability (high, low) as factors. There was no main effect of strength on *R*^2^, *F*(1, 59) = 0.45, *p* = .51, $\eta_{p}^{2}$ = .01, BF = 0.17. However, there was a significant effect of item characteristic variability on *R*^2^, *F*(1, 59) = 47.32, *p* < .001, $\eta_{p}^{2}$ = .46, BF = 1.59 × 10^9^, and no interaction, *F*(1, 59) = 0.33, *p* = .57, $\eta_{p}^{2}$ = .01, BF = 0.23. This indicates that the proportion of variance in the ratings explained by the predictor variables increased because of our item characteristic manipulation and not our strength manipulation. *R*^2^ was on average 5% to 6% greater in the high variability conditions than the low ones.

#### Parameter estimates

All mean UVSD model parameter estimates for each condition are found in Table 3. To compare the influence of our manipulations upon parameter estimates of mean strength from the UVSD model, we conducted a 2 × 2 ANOVA on *d* with strength (high, low) and item characteristic variability (high, low) as factors. There was a large main effect of the strength manipulation *d*, *F*(1, 59) = 42.56, *p* < .001, $\eta_{p}^{2}$ = .42, BF = 2.07 × 10^10^. There was no effect of item characteristic variability on *d*, *F*(1, 59) = 0.63, *p* = .43, $\eta_{p}^{2}$ = .01, BF = 0.16, and no interaction was present, *F*(1, 59) = 0.45, *p* = .50, $\eta_{p}^{2}$ = .01, BF = 0.23.

**Table 3.** Mean parameter estimates (standard deviations in brackets) output by the UVSD model, per condition, in Experiments 1 and 2. Mean values of σ_o_ were calculated using the log scale and then exponentiated.

Experiment	Parameter	Condition			
		High strength, High variability	High strength, Low variability	Low strength, High variability	Low strength, Low variability
1	*d*	1.50 (0.76)	1.42 (0.79)	0.92 (0.57)	0.92 (0.71)
	σ_o_	1.43 (1.32)	1.36 (1.33)	1.27 (1.29)	1.28 (1.27)
	*C*_1_	–0.94 (1.13)	–1.19 (1.60)	–1.30 (1.39)	–1.76 (2.49)
	*C*_2_	–0.02 (1.16)	–0.13 (1.12)	–0.24 (0.88)	–0.51 (1.90)
	*C*_3_	0.72 (0.45)	0.56 (0.48)	0.51 (0.43)	0.42 (0.41)
	*C*_4_	1.24 (0.61)	1.07 (0.67)	1.11 (0.76)	1.06 (0.85)
	*C*_5_	1.90 (1.04)	1.77 (1.06)	1.93 (1.05)	1.86 (1.21)
2	*d*	1.87 (1.42)	1.42 (1.15)	0.89 (0.51)	0.80 (0.54)
	σ_o_	1.53 (1.42)	1.29 (1.41)	1.26 (1.24)	1.22 (1.23)
	*C*_1_	–1.21 (3.40)	–1.04 (1.86)	–1.16 (1.60)	–1.31 (1.75)
	*C*_2_	0.01 (2.26)	–0.24 (1.84)	–0.26 (1.24)	–0.31 (1.31)
	*C*_3_	0.75 (0.48)	0.52 (0.45)	0.43 (0.37)	0.37 (0.45)
	*C*_4_	1.26 (0.66)	1.02 (0.57)	0.97 (0.46)	0.86 (0.52)
	*C*_5_	1.93 (0.80)	1.76 (0.67)	1.69 (0.51)	1.72 (1.04)

The ordinal pattern of σ_o_ across conditions followed that of *d*. Another 2 × 2 ANOVA with strength and item characteristic variability as factors was conducted with σ_o_ as the dependent variable. A significant main effect of strength was found, *F*(1, 59) = 6.03, *p* = .017, η_p_^2^ = .10, BF = 6.03. Again, there was no effect of item characteristic variability, *F*(1, 59) = 0.49, *p* = .49, η_p_^2^ = .01, BF = 0.20, and no significant interaction, *F*(1, 59) = 0.94, *p* = .34, η_p_^2^ = .02, BF = 0.29. This is evidence that estimates of σ_o_ were determined by mean memory strength, rather than encoding variability from our manipulated item characteristics.

#### Curve-fitting analysis

As an exploratory analysis, we fitted linear and polynomial models to estimates of *d* and σ_o_ to determine the shape of the function by which σ_o_ scales with *d*. We evaluated three scaling formulae; one in which scaling is linear (σ_o_ = *y* + *bd*, where *y* is the intercept), one with linear and quadratic components (σ_o_ = *y* + *b*_1_*d* + *b*_2_*d*^2^), and one with linear, quadratic, and cubic components (σ_o_ = *y* + *b*_1_*d* + *b*_2_*d*^2^ + *b*_3_*d*^3^). In a sequential regression procedure, each model was fit to data, and the difference in the fit of each model was computed sequentially using frequentist and Bayesian ANOVAs. Linear models with intercepts between 0.02 and 0.09 and coefficients between .17 and .23 tended to fit the data best (see Table 4). In all conditions, there was no reliable improvement in fit being evident in frequentist ANOVAs from adding quadratic, or quadratic and cubic components (*p*s > .28). Bayesian ANOVAs also supported this conclusion (BFs < 0.44).

**Table 4.** Best fitting regression models relating mean strength and old item variance in each experiment, with *R*^2^ values.

Experiment	Condition	Best fitting model	*R* ^2^
Experiment 1	High Strength, High Variability	σ_o_ = 0.09 + 0.18(*d*)	.23
	High Strength, Low Variability	σ_o_ = 0.05 + 0.18(*d*)	.24
	Low Strength, High Variability	σ_o_ = 0.02 + 0.23(*d*)	.28
	Low Strength, Low Variability	σ_o_ = 0.09 + 0.17(*d*)	.26
Experiment 2	High Strength, High Variability	σ_o_ = 0.08 + 0.18(*d*)	.56
	High Strength, Low Variability	σ_o_ = −0.04 + 0.21(*d*)	.50
	Low Strength, High Variability	σ_o_ = 0.06 + 0.19(*d*)	.20
	Low Strength, Low Variability	σ_o_ = 0.11 + 0.11(*d*)	.09
Experiment 3	Old, High Variability	σ_oh_ = 0.01 + 0.23(*d*)	.32
	Old, Low Variability	σ_ol_ = −0.01 + 0.19(*d*)	.23

### Discussion

We found no evidence that varying item characteristics influenced estimates of old item variance, σ_o_, despite our item characteristic variance manipulation having a clear impact on recognition confidence ratings. Instead, overall memory strength determined estimates of σ_o_. Moreover, curve-fitting analyses showed a positive, linear association between *d* and σ_o_, further providing evidence of an association between strength and old item variance. These results provide clear evidence that a strength scaling trend can explain the old item variance in the present experiment, with no reliable contributions of encoding variability being observed as a result of our item characteristic manipulation.

Although the effect of our item characteristic manipulation on recognition confidence ratings was significant, this effect was of small to medium size (Cohen, 1988; see Table 2 for *R*^2^ values from each condition). It is therefore possible that, even if our manipulation was representative of encoding variability, it still might not have translated to differences in σ_o_ that were detectable. This outcome would be unable to explain the presence of the currently observed strength scaling trend; however, it would mean that the encoding variability hypothesis might also hold under a stronger manipulation. In Experiment 2, we aim to establish such a manipulation by adding even more variability to the characteristics of old items than in Experiment 1.

## Experiment 2

Although variability in item characteristics affected recognition confidence responses in Experiment 1, it is possible that the strength of this manipulation was constrained by the Gaussian distributional assumption by which item characteristic variables were sampled. This assumption was driven by the specification of the encoding variability hypothesis, which states that added strength is Gaussian (Jang et al., 2012). Although this assumption is plausible, the Lyapunov central limit theorem states that many non-identical independent random variables can still sum to a Gaussian form, provided they satisfy certain mathematical assumptions. In practice, it is hard to verify these assumptions since memory strength is a latent variable. However, it is possible that adding non-Gaussian strength distributions may result a product that is at least close to the Gaussian old item distribution in the UVSD. To this end, Experiment 2 will follow a method similar to Experiment 1, although the distributions of item characteristic values will be permitted to be non-Gaussian. This will maximise the variability of item characteristics even more than in Experiment 1, thereby increasing the chance of a detectable effect of encoding variability. If this manipulation is successful, the same predicted outcomes from Experiment 1 apply.

### Methods

#### Participants

In all, 64 participants (16 males, 47 females) with a mean age of 22.8 (*SD* = 10.7) from the University of Plymouth Psychology Participation Pool took part in this experiment in exchange for either £8 or course participation points. Each participant spoke English fluently as a first language, was not dyslexic, and had not participated in Experiment 1. Participants were either University of Plymouth psychology undergraduates, or members of the public from the Plymouth area.

#### Materials and procedure

Stimuli were 480 words (60 old and 60 new in each condition). These words were sampled with the same constraints as in the previous experiment, with only the following differences:

1. The requirement for the distributions of word frequency, concreteness, and AOA scores to not significantly deviate from a Gaussian in the high encoding variability lists was removed. Instead, the distributions did not strictly adhere to any preset distributional shape and were only constrained to be roughly symmetrical. This was achieved by scoring each word by a weighted index of word frequency, concreteness, and AOA scores, and grouping words based on their distance from the mean of the index, measured in standard deviations. Words were then randomly sampled in equal quantities from each group, resulting in distributions of each encoding variable that were non-Gaussian and had more variance than in Experiment 1.
2. Due to the sampling method, the correlations between item characteristics were stronger, despite no threshold correlation values being imposed as generative constraints. The negative correlations between word frequency and concreteness ranged between *r* = −.89 and *r* = .92. The negative correlations between word frequency and AOA were between *r* = −.60 and *r* = −.77. The positive correlations between concreteness and AOA were between *r* = .50 and *r* = .65.

The strength manipulation and other materials were identical to the previous experiment. The procedure was also identical to that of Experiment 1, with the only difference being that new word lists replaced those that were previously used.

### Results

We excluded four participants who used the “Sure New” and “Sure Old” responses in nearly all test phase trials. These exclusions were made for the same reasons as those in Experiment 1. We also analysed the natural logarithm of σ_o_, as in the previous experiment.

#### Study task performance

As in Experiment 1, the mean proportion of correct responses in the “Low Strength, High Variability” condition (*M* = .92, *SE* = .01) was not significantly different from that in the “Low Strength, Low Variability” condition (*M* = .93, *SE* = .01), *t*(59) = −0.31, *p* = .76, 95% CI [−0.04, 0.03], BF = 0.15.

#### Item characteristic variability manipulation

Our item characteristic variability manipulation was assessed using the same multiple regression analysis as in Experiment 1. The proportion of significant regression models and mean *R*^2^ values for each condition are reported in Table 2. A 2 × 2 ANOVA on *R*^2^ with strength and item characteristic variability as factors found no main effect of strength on *R*^2^, *F*(1, 59) = 0.79, *p* = .38, $\eta_{p}^{2}$ = .01, BF = 0.17. There was, however, a significant effect of item characteristics on *R*^2^, *F*(1, 59) = 45.21, *p* < .001, $\eta_{p}^{2}$ = .43, BF = 5.37 × 10^12^ and no interaction, *F*(1, 59) = 0.61, *p* = .44, $\eta_{p}^{2}$ = .01, BF = 0.24. As in Experiment 1, this indicates that the proportion of variance in recognition confidence ratings accounted for by the predictor variables increased between 7% and 8% with our item characteristic variability manipulations, and not our strength manipulation.

#### Parameter estimates

Mean parameter estimates for Experiment 2 are presented in Table 3; 2 × 2 ANOVAs were conducted to determine whether our variability or strength manipulations influenced estimates of *d*. There was a significant main effect of strength on *d*, *F*(1, 59) = 41.96, *p* < .001, $\eta_{p}^{2}$ = .42, BF = 6.68 × 10^9^. There was also a significant main effect of item characteristic variability, although this was accompanied by an inconclusive Bayes Factor, *F*(1, 59) = 9.98, *p* = .003, $\eta_{p}^{2}$ = .15, BF = 1.63. There was not a significant interaction, *F*(1, 59) = 3.70, *p* = .06, $\eta_{p}^{2}$ = .06, BF = 0.79. Our strength manipulations were therefore shown to have a main effect on *d*, however, there was some weak evidence of an effect of item characteristic variability as well. This suggests that our non-Gaussian item characteristic manipulation may have had some unintended effect upon memory strength.

To assess whether variability in item characteristics or overall strength influenced estimates of σ_o_, we conducted a 2 × 2 ANOVA. There was a significant main effect of strength on σ_o_, *F*(1, 59) = 11.99, *p* = .001, $\eta_{p}^{2}$ = .17, BF = 46.77. There was also a significant effect of item characteristic variability on σ_o_, *F*(1, 59) = 11.16, *p* = .001, $\eta_{p}^{2}$ = .16, BF = 7.00. There was also no significant interaction, *F*(1, 59) = 3.22, *p* = .08, $\eta_{p}^{2}$ = .05, BF = 1.22. In sum, there was strong evidence for both an effect of strength and item characteristic variability on σ_o_.

#### Curve-fitting analyses

We conducted the same curve-fitting analyses as in the previous experiment; results from this analysis are found in Table 4. Linear models fitted best in all conditions, as quadratic and cubic components did not improve model fit (*p*s > .18, BFs < 0.70).

### Discussion

Unlike in Experiment 1, there was evidence for main effects of both item characteristics and overall strength on estimates of σ_o_ in Experiment 2. However, contrary to the aims of our study, our manipulation of item characteristic variability significantly affected estimates of *d*, though the Bayes Factor for this result was inconclusive. It is therefore difficult to judge whether some effect of our item characteristic manipulation on σ_o_ was the genuine result of increased encoding variability, or a consequence of the manipulation also affecting memory strength. What is clearer is that our manipulation of memory strength influenced both estimates of *d* and σ_o_, and that this is not explicitly accounted for by the current specification of the encoding variability hypothesis.

It is possible that our non-Gaussian item characteristic variability manipulation gave rise to the unexpected effects of item characteristic variability on *d*. Although we aimed to sample words with roughly symmetrical distributions of word frequency, concreteness, and AOA values, it is possible that deviating from a Gaussian form caused the distributions of these item characteristics to be less symmetrical than those in Experiment 1. This could have resulted in our manipulation having unintended effects on old item memory strength, shifting the value of *d* as well as affecting σ_o_. Indeed, our variability manipulation in Experiment 1 did not have unexpected effects on *d* as well as σ_o_, despite the only major difference between each experiment being the distributional assumption by which words were sampled. In any case, it is still more certain that overall memory strength has a substantial effect on estimates of σ_o_ than our manipulation of item characteristics in this experiment.

## Experiment 3

In our present methods (and those in Spanton & Berry, 2020), we matched the level of manipulated variability in item characteristics across the old and new word lists in a test phase. Specifically, in Experiments 1 and 2, the old and new words in each test phase had very similar high or low variability in terms of word frequency, concreteness, and AOA. This decision was made to minimise the chance that participants would use differences in item characteristics in each list as an additional memory cue, which would confound our manipulations. We assumed that in these methods, σ_o_ would still be greater in high variability conditions if the encoding variability hypotheses were to be true. However, it is important to note that σ_o_ is a ratio of target-lure variance. This is the case regardless of the fixed value of σ_n_, although we fixed it to equal 1 to ensure the target-lure ratio and the absolute value of σ_o_ were equal. Consequently, if our manipulation of item characteristic variance affected σ_n_, it is possible that in our previous experiments, any effect of old item characteristic variability on σ_o_ may have been offset by the high, matched level of variance of new item strength. This would lower the chance of observing an encoding variability effect.

To circumvent this issue while mitigating the possibility that item characteristics in each old or new word list might serve as an additional memory cue, we can design an experiment with a single study/test phase. Within this phase, half of the old and new items can have highly variable item characteristics (word frequency, concreteness, AOA, and word length), whereas the other conditions can have low variance in their item characteristics. Overall memory performance and variability in memory for these four stimulus conditions (“old-high,” “old-low,” “new-high,” “new-low”) can be analysed separately. However, because the complete old and new word lists share the same overall variability in item characteristics, participants cannot use differences in these characteristics as a cue to aid their recognition judgements. If the variance in memory strength for each condition is modelled on a participant level, the encoding variability hypothesis would predict that this variance would be greater in the old-high condition (σ_oh_) than in the old-low condition (σ_ol_).

**Figure 1.**
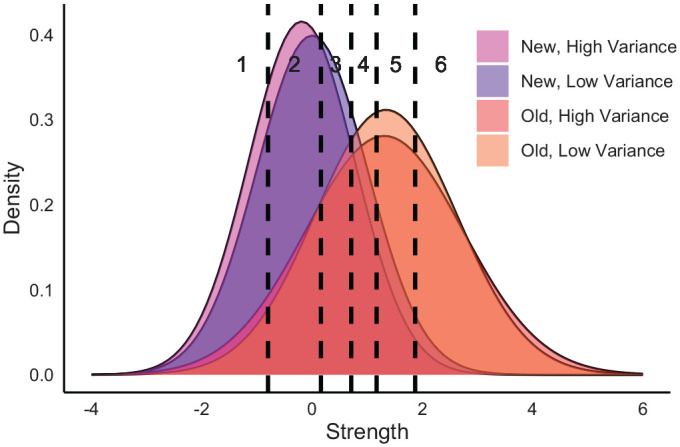
A depiction of our extended UVSD model specification, with parameters set to the mean estimates recovered from Experiment 3. UVSD: unequal variance signal detection.

To allow the estimation of the key parameters in this experiment, we must define four distributions in the UVSD model—one for each condition (see Figure 1). The mean and standard deviation of the new-low distribution can be fixed so that μ_nl_ = 0 and σ_nl_ = 1, allowing the means and standard deviations of each other condition to be free and scaled upon these fixed parameters. Since all the conditions appear to the participant in one study-test phase, it follows that the same decision criteria should be used to model judgements for words in every sub-list. Extending the UVSD model to represent this design therefore requires the free parameters θ = {μ_nh_, μ_ol_, μ_oh_, σ_nh_, σ_ol_, σ_oh_, *C*_1_, *C*_2_, . . . *C_I_*}. The specification of this model extension, alongside its likelihood function and parameter recovery simulations, can be found in Appendix A.

### Method

#### Participants

In all, 75 undergraduate psychology students from the University of Plymouth (57 females, 16 males, 2 non-binary/other) completed the experiment in exchange for course credits. Three participants were excluded during analyses due to outlying parameter estimates (see Results), leaving an effective sample of 72 participants that allowed for detection of a minimum effect size *d*_z_ = 0.33 at 80% power in a paired samples *t*-test.

#### Materials

The stimuli consisted of the first two lists of high variability words and low variability words from Experiment 1. The experiment was implemented using the OSWeb functionality of OpenSesame (Mathôt et al., 2012). Participants completed the task in a lab, using Lenovo desktop computers running a browser window containing the experiment programme.

#### Procedure

Participants first completed a study phase consisting of 120 trials. In each trial, they viewed a fixation point for 500 ms, a word for 3000 ms, and an inter-trial interval (a blank screen) for 500 ms. The words in the study phase were made up of one set of 60 high variability words, and one set of 60 low variability words; these sets were intermixed and presented in a different random order for each participant. The allocation of each high and low variability word list as old or new item was also randomised across participants. Participants were instructed to pay attention to each word during the study phase, and that they should try to remember as many words as possible for a later memory test. After the study phase, participants had a 60 s break before reading instructions for the test phase.

The test phase had the same trial level structure as those in Experiments 1 and 2, with participants making recognition judgements on the same 1–6 scale. A total of 240 words were presented (120 old, 120 new), with the new words consisting of the remaining high and low item variability lists. As in the study phase, each participant completed a different random order of trials. Upon completing the test phase, participants input their age and gender into the experimental programme before reading a full debrief.

### Results

Three participants were excluded from all analyses for having outlying parameter estimates, in line with the approach taken in Experiments 1 and 2. We also log transformed the parameters σ_oh_, σ_ol_, and σ_nh_ in line with our approach in Experiments 1 and 2. Bonferroni corrections were applied to all pairwise comparisons.

#### Item characteristic variability manipulation

As in Experiments 1 and 2, regression analyses were conducted to gauge the effect of each manipulated item characteristic on recognition confidence responses. Each participant’s data were split by item type (old, new) and item characteristic variability level (high, low), and regression models with word frequency, concreteness, AOA, and word length as predictors were fit to each combination of factors. The proportion of significant regression models and mean *R*^2^ values can be found in Table 2. A 2 × 2 within-subjects ANOVA on *R*^2^ was then conducted with item type and item characteristic variability level as factors. This ANOVA revealed a significant main effect of item characteristic variability on *R*^2^, *F*(1, 71) = 18.67, *p* < .001, $\eta_{p}^{2}$ = .21, BF = 1018.28. There was no significant main effect of item type, *F*(1, 71) = 0.27, *p* = .61, $\eta_{p}^{2}$ < .01, BF = 0.15, and no significant interaction, *F*(1, 71) = 2.33, *p* = .13, $\eta_{p}^{2}$ = .03, BF = 0.50. This indicates that variance in recognition confidence ratings was explained by our item characteristic variability manipulation, rather than the presence of words in the study phase. The high variability words accounted for around 4% more total variance in recognition confidence ratings for old items than low variability words, which is a roughly comparable increase with Experiments 1 and 2.

#### Parameter estimates

**Figure 2.**
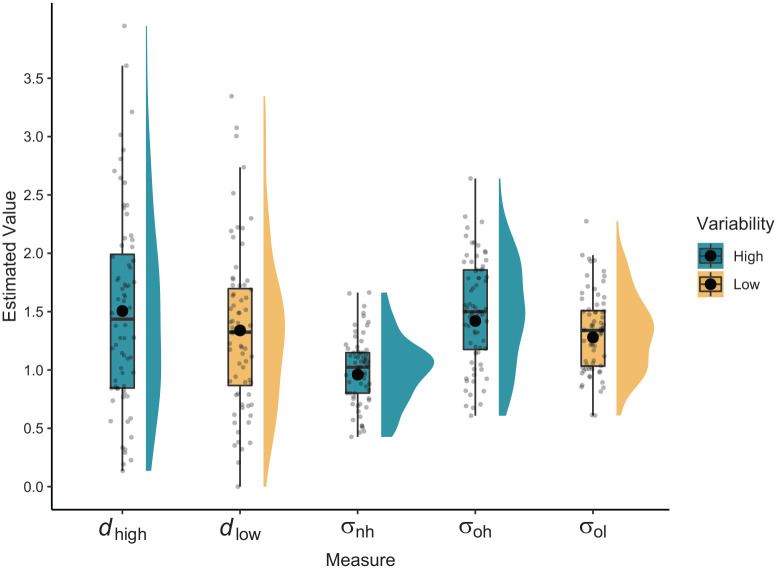
Raincloud plots of σ free parameter estimates and discriminability measures, with circular points denoting means. The mean of each log-sigma parameter was calculated, then exponentiated.

The parameter estimates from the UVSD model can be found in Table 5. A one-factor repeated measures ANOVA with a Greenhouse–Geisser sphericity correction was used to compare the estimates of σ in the old-high, old-low, and new-high conditions. Estimates significantly differed across conditions, *F*(1.66, 117.91) = 85.66, *p* < .001, $\eta_{p}^{2}$ = .55, BF = 1.75 × 10^10^. The ordinal pattern of variance estimates for each distribution can be seen in Figure 2. Pairwise comparisons confirmed that estimates of σ_nh_ were reliably lower than those of both σ_oh_, *t*(71) = −11.33, *p* < .001, BF = 8.27 × 10^14^, and σ_ol_, *t*(71) = −9.00, *p* < .001, BF = 2.42 × 10^10^. Crucially however, estimates of σ_oh_ were significantly greater than estimates of σ_ol_, *t*(71) = 4.06, *p* < .001, BF = 300.76. Furthermore, a one-sample *t*-test also revealed that σ_nh_ did not significantly differ from 1, the fixed value of σ_nl_, *t*(71) = −1.26, *p* = .21, 95% CI: [−0.11, 0.03], BF = 0.28. This means that our manipulation of item characteristic variability only affected old items, in line with the encoding variability hypothesis.

**Table 5.** Mean parameter estimates for the UVSD model in Experiment 3 (standard deviations in brackets).

Parameter	*M*	*SD*
μ_nh_	–0.19	(0.28)
μ_ol_	1.34	(0.69)
μ_oh_	1.31	(0.77)
σ_nh_	0.96	(1.36)
σ_ol_	1.28	(1.31)
σ_oh_	1.42	(1.40)
*C*_1_	–0.80	(1.22)
*C*_2_	0.16	(0.60)
*C*_3_	0.71	(0.44)
*C*_4_	1.17	(0.56)
*C*_5_	1.87	(0.70)

Note: The fixed parameters are not shown here: μ_nl_ was fixed to 0, and σ_nl_ was fixed to 1. The mean and standard deviation of each log-transformed sigma parameter was calculated, then exponentiated.

UVSD: unequal variance signal detection.

To assess the possibility that differences in old item variance may have been driven by effects of overall memory strength, we calculated discriminability (*d*) measures for high and low variability conditions. These measures were given by calculating *d*_high_ = µ_oh_ − µ_nh_ and *d*_low_ = µ_ol_ − µ_nl_, respectively, on a participant level. Discriminability measures were reliably greater for high variability items than for low variability items, *t*(71) = 2.90, *p* = .004, 95% CI [0.05, 0.28], BF = 6.05. This increase in discriminability for high variability items was likely driven by estimates of μ_nh_ being reliably lower than 0, the fixed value of μ_nl_, *t*(71) = −5.72, *p* < .001, 95% CI [−0.26, −0.12], BF = 58,633.94. By contrast, no reliable differences were found between estimates of μ_oh_ and μ_ol_, *t*(71) = −0.60, *p* = .55, 95% CI [−0.11, 0.06], BF = 0.15. This means that greater overall memory strength for high variability items coincided with greater estimates of old item variance for those items. Therefore, as in Experiment 2, we cannot conclude whether increases in old item variance are due to manipulated encoding variability, because these increases were not independent of changes in overall strength.

For comparison with the results of Experiments 1 and 2, and those of Spanton and Berry (2020), we examined the relationship between discriminability and σ_o_ parameters within the high and low variability conditions using linear regression (see Table 4). There was a significant positive relationship between *d*_high_ and σ_oh_, *F*(1, 70) = 32.64, *p* < .001, *R*^2^ = .32. There was also a significant positive relationship between *d*_low_ and σ_ol_, *F*(1, 70) = 21.07, *p* < .001, *R*^2^ = .23. This indicates that estimates of mean memory strength and variability in memory strength for old items were positively associated.

### Discussion

The present results showed a clear increase in estimates of old item variance in high variability conditions while estimates of new item variance remained constant across high and low variability conditions. However, this selective increase in old item variance cannot be taken as clear evidence for the encoding variability hypothesis, because old and new items were also more discriminable in the high variability conditions. Therefore, as in Experiment 2, the effect of our item characteristic variability manipulation coincided with simultaneous increases in memory strength. Moreover, measures of discriminability and old item variance were positively associated on a participant level in both high and low variability conditions, mirroring the linear relationships found between σ_o_ and *d* in Experiments 1 and 2.

Although we saw unexpected simultaneous effects on memory strength and old item variance in this experiment, our manipulation was at least successful in increasing old item variance while controlling new item variance. This shows that the present experimental design has promise in providing a principled test of the encoding variability hypothesis that is more certain to affect the ratio of old/new item variance than our Experiments 1 and 2. The difference in memory strength for high and low variability items in this experiment was unexpected given that the same stimuli did not by themselves elicit such a difference when used in Experiment 1. However, these results show (along with previous attempts) that it is often hard to manipulate encoding variability in a theoretically principled way without the presence of additional confounds. The present single-block design appears to be a promising way of selectively manipulating old item variance, but effects on discriminability should also be considered in future experiments with this design.

## General discussion

Although it has been suggested that encoding variability causes the old item variance effect (Wixted, 2007), previous research has suggested that it cannot solely account for the UVSD model’s unequal variance assumption (Koen et al., 2013; Spanton & Berry, 2020). Our results from Experiment 1 reiterate this conclusion, showing that σ_o_ tends to be determined by mean strength (*d*) in a linear scaling function, with no main effect of varying item characteristics. In Experiment 2, there was a main effect of varying item characteristics on σ_o_, though this was partially confounded by a weaker effect of item characteristic variability on *d*, and overall strength still had the greatest influence on σ_o_ in this experiment. Experiment 3 showed that increasing item characteristic variability resulted in a selective increase in old item variance while new item variance remained constant. However, this once again coincided with increased discriminability measures in high variability conditions. Positive participant-level associations between old item variance and memory strength measures persisted across all experiments. We therefore conclude that overall memory strength can determine old item variance estimates in the UVSD model independently of encoding variability. By contrast, any effects of encoding variability prompted by varying item characteristics at study are not fully separable from increases in mean memory strength in the present experiments.

It has previously been stated that manipulating encoding variability by varying item characteristics would be very challenging to achieve on theoretical grounds (Spanton & Berry, 2020). The present results reiterate this conclusion. Despite manipulating multiple item characteristics at once to achieve a meaningful effect on recognition confidence ratings—in this instance, one that causes an *R*^2^ difference between conditions corresponding to a small to medium effect size (Cohen, 1988)—this did not lead to clear increases in old item variance. Moreover, it proved difficult to manipulate mean memory strength orthogonally without confounds, as seen in Experiment 2. In Experiment 3, we were able to contribute a new manipulation that affected the old/new item variance ratio but were unable to control mean memory strength to observe an unequivocal encoding variability effect, despite using stimuli from Experiment 1 that did not previously elicit confounding effects on mean strength. In sum, these experiments emphasise that the encoding variability hypothesis is difficult to test. Further, no conclusive evidence of its independent contribution to the old item variance effect has yet been found.

Although we did not find unambiguous support for encoding variability, our results showed that mean strength manipulations can independently increase old item variance estimates on a group level, and that these parameter estimates have a positive linear association on a participant level. These findings align with previous reports of the *z*-ROC slope decreasing as memory performance increases (Glanzer et al., 1999; Parks & Yonelinas, 2007), lending further support to the existence of a strength scaling trend. Based on this, it is unlikely that encoding variability alone can explain changes in old item variance in the UVSD model. It is possible that the specification of the encoding variability hypothesis suggested by Jang et al. (2012) can be extended to include strength scaling by assuming that the mean of the added strength distribution (μ_A_) scales with the variance of added strength (σ_A_) in the equation *O* = *B* + *A*. However, this extension would not address other limitations of the encoding variability hypothesis identified by Spanton and Berry (2020). It would also not account for the effect of retrieval manipulations on estimates of old item variance in the UVSD model (Koen et al., 2013). As such, it is likely that this hybrid specification would not give a satisfactory explanation of the old item variance effect.

Although our results pertain foremost to the UVSD model, they raise the broader question of how other signal detection models and theoretical frameworks represent encoding variability and strength scaling. Despite finding no conclusive evidence for the encoding variability hypothesis specified by Jang et al. (2012) in relation to the UVSD model, we do not dispute the general idea that some items are encoded more strongly than others. This is almost certainly true, and so it is useful to consider how models that include different psychological processes or even explicit mathematical representations of how information is stored, retained, and retrieved, account for this. Doing so may provide useful information that could feed back into shaping a more valid explanation of the old item variance effect in the UVSD model. We now consider some alternative models and theoretical explanations for encoding variability and the strength scaling trend.

There are many other signal detection models that could represent encoding variability and strength scaling. For instance, both the dual-process signal detection (DPSD; Yonelinas, 1994) and mixture signal detection (DeCarlo, 2002) models can account for increases in old item variance with changes in their parameters (Spanton & Berry, 2020). The DPSD model also predicts that an increase in the probability of recollection for old items boosts memory strength and old item variance. Without ruling out the contribution of factors during retention and retrieval, this gives a meaningful interpretation of the strength scaling trend that could be tested empirically with a manipulation of recollection. It is also worth noting that alongside these commonly used models, there are a wide variety of other possible signal detection models with different, non-Gaussian memory strength distributions, (DeCarlo, 1998; Malejka & Bröder, 2019). These models could provide substantively different interpretations of trends in data due to their mathematical specifications, the implications of which should also be investigated.

Models outside of the signal detection framework that include explicit representations of items in memory could also be studied regarding encoding variability and strength scaling. Global matching models such as SAM (Gillund & Shiffrin, 1984) and MINERVA 2 (Hintzman, 1988) predict that representations can vary due to factors at encoding, retention, and retrieval, in contrast to the encoding variability hypothesis. While these models can account for data in which old item variance scales with memory strength, both can also represent opposing trends, such as decreased variability in memory with increased performance, with plausible parameter behaviour. Due to this behaviour and the nature of their representations of feature-level memory for items, models such as these could also provide a broader theoretical perspective on the encoding variability hypothesis and the strength scaling trend.

It also stands that changes in the shape of the *z*-ROC are not exclusively caused by mnemonic factors (Malmberg & Xu, 2006; Rabe et al., 2021), and many models reflect this. For instance, previous models have added variability to recognition decision criteria, and have potential to add to discussion about the unequal variance assumption (Benjamin et al., 2009). Although the addition of equal variability to all decision criteria does not affect the *z*-ROC slope (Wickelgren, 1968), it has been shown that forms of selective criterion variability can (Cabrera et al., 2015). However, such variability may also decrease discriminability depending upon its form, putting such an effect in opposition to a scaling trend. It has also been shown that more accurate estimates of mean strength and old item variance can be obtained using models with variable criteria (Cabrera et al., 2015). Using variable criterion models may therefore inform whether strength scaling is seen in models that differentiate between decision processes and underlying mnemonic representations. Furthermore, RTCON and Diffusion models (Osth et al., 2017; Ratcliff & Starns, 2009, 2013), give lower estimates of old item variance in comparison to *z*-ROC slopes from signal detection models, prompted in part by changes in non-mnemonic sources of trial-to-trial variability. The extent to which explanations of encoding variability align with reaction time distributions from the RTCON models that support an unequal variance assumption could also be investigated in the future, with non-mnemonic factors in mind.

These models could help to establish the generality of the strength scaling trend and determine why some manipulations of strength do not change the slope of the *z*-ROC. Early work on the topic proposed that although the *z*-ROC slope is commonly less than 1, memory strength manipulations do not affect its supposed value of ~0.8 (Ratcliff et al., 1992, 1994). Although Glanzer et al. (1999) later presented substantial evidence demonstrating that *z*-ROC slopes are not constant, their analyses of previous studies that used stimulus repetition to manipulate memory strength found that the *z*-ROC slope consistently remained unchanged despite significant increases in strength. Similarly, although Yonelinas (1994) found a strength scaling trend when manipulating study list length, they found constant *z*-ROC slopes when increasing memory strength with a study time manipulation. Changes in memory performance also did not provoke changes in the *z*-ROC slopes in some later studies (Grider & Malmberg, 2008; Starns et al., 2012). Understanding these results while considering alternative models and theoretical explanations may provide information about the causes and boundary conditions of the strength scaling trend in Gaussian signal detection models.

To conclude, we investigated whether changes in the variance of recognition memory strength for old items in the UVSD model were prompted by manipulations of mean strength during study, or the variability of item characteristics. We found evidence that levels of overall memory strength influenced old item variance in Experiment 1, with no contribution of varying item characteristics. A main effect of overall strength was also found in Experiment 2; there was also a main effect of our item characteristic manipulation, however this was partially confounded by a simultaneous effect on memory strength. Experiment 3 again showed that increases in old item variance coincided with simultaneous increases in memory strength in an experimental design with a single study/test block. These results show that while mean memory strength can independently determine estimates of old item variance in the UVSD model, there is still no clear evidence for a contribution of encoding variability without a simultaneous increase in mean strength. We recommend the use of new theoretical perspectives to further examine these trends and their implications for the UVSD model, and for our understanding of recognition memory more broadly.

## Supplemental Material

sj-docx-1-qjp-10.1177_17470218221136498 – Supplemental material for Does variability in recognition memory scale with mean memory strength or encoding variability in the UVSD model? Click here for additional data file. Supplemental material, sj-docx-1-qjp-10.1177_17470218221136498 for Does variability in recognition memory scale with mean memory strength or encoding variability in the UVSD model? by Rory W Spanton and Christopher J Berry in Quarterly Journal of Experimental Psychology
