# Supplementary material for: Does variability in recognition memory scale with mean memory strength or encoding variability in the UVSD model?
Source: Q J Exp Psychol (Hove). 2022 Nov 21;76(9):2037–52. doi: 10.1177/17470218221136498 (PMC10467009; doi:10.1177/17470218221136498)
Supplement: sj-docx-1-qjp-10.1177_17470218221136498 – Supplemental material for Does variability in recognition memory scale with mean memory strength or encoding variability in the UVSD model? [file sj-docx-1-qjp-10.1177_17470218221136498.docx]

Supplementary Material for:

**Does variability in recognition memory scale with mean memory strength or encoding variability in the UVSD model?**

Rory W. Spanton^1^ & Christopher J. Berry^1^

^1^School of Psychology, University of Plymouth

**Supplementary Material A: Parameter Recovery Simulation for Experiment 3**

A total of 11 free parameters are required to simultaneously model the four conditions in Experiment 3 with our extended UVSD model specification. This is four more than was required to model each test phase in Experiments 1 and 2, and it is therefore important to determine whether reliable estimates of the free parameters can be recovered. To do this, 75 datasets with the same number of trials as data from Experiment 3 (240 total) were simulated from, and the UVSD model was fit to these datasets to recover the true generative parameters. The datasets were generated using the UVSD model’s parameter specification; for each free parameter, values were drawn from a uniform distribution to evenly cover a plausible parameter space. The bounds on these uniform distributions and the mean values of the true and estimated parameters can be found in Table A1. The bounds upon the criteria distributions were defined in a way that ensured each successive criterion was greater than the last.

Bayesian *t*-tests found a strong absence of a difference between estimated and true values for all the model parameters (BFs ≤ 0.33) except for μ_oh_, μ_ol_, σ_ol_, where inconclusive evidence was found, although these Bayes Factors were still relatively low. This indicates that the model can successfully estimate true parameter values in simulated data with the same number of trials and participants as in Experiment 3. It is of note that some outlying estimates were produced in this simulation, which reflect in the mean and standard deviation of certain parameter estimates. Although these outliers make the means less representative of the model’s general predictions about data (and are therefore excluded in our experiments), the model can still produce a generally accurate account of the true parameters in simulated data with these outliers included.

Table A1.

*Generative lower and upper bounds* a *and* b *on the uniform distributions used to simulate true parameters, the means of the true and estimated parameters given by the extended UVSD model (SDs in parentheses), and the Bayes Factor of the comparisons between true and estimated parameter values.*

| Parameter | *a* | *b* | True Mean | | Mean Estimate | | BF |
| --- | --- | --- | --- | --- | --- | --- | --- |
| μ_nh_ | -1 | 1 | -0.08 | (0.56) | -0.02 | (0.73) | 0.20 |
| σ_nh_ | 0.5 | 4 | 2.30 | (0.98) | 2.50 | (1.43) | 0.27 |
| μ_oh_ | 0 | 4 | 2.14 | (1.08) | 3.12 | (6.59) | 0.37 |
| σ_oh_ | 0.5 | 4 | 2.45 | (1.02) | 2.56 | (1.45) | 0.20 |
| μ_ol_ | 0 | 4 | 1.94 | (1.19) | 2.93 | (5.71) | 0.47 |
| σ_ol_ | 0.5 | 4 | 2.33 | (1.03) | 2.66 | (1.90) | 0.40 |
| *C*_1_ | -1 | 0.2 | -0.42 | (0.35) | -0.45 | (0.43) | 0.20 |
| *C*_2_ | *C*_1_ + 0.01 | *C*_1_ + 1 | 0.09 | (0.42) | 0.10 | (0.48) | 0.18 |
| *C*_3_ | *C*_2_ + 0.01 | *C*_2_ + 1 | 0.61 | (0.53) | 0.63 | (0.58) | 0.18 |
| *C*_4_ | *C*_3_ + 0.01 | *C*_3_ + 1 | 1.18 | (0.58) | 1.23 | (0.67) | 0.20 |
| *C*_5_ | *C*_4_ + 0.01 | *C*_4_ + 1 | 1.68 | (0.64) | 1.75 | (0.75) | 0.21 |

c

**Maximum Likelihood Estimation Fit Procedure**

As in the rest of our experiments, the UVSD model was fit to data using maximum likelihood estimation. To fit the model to a given participant’s data in Experiment 3, initial starting estimates of each of the model’s free parameters were derived from the data. The parameters were derived using the approximations detailed in the supplemental materials of Spanton and Berry (2020), with *dʹ* being used to approximate both old item distribution means, and 1 / *z*-ROC slope used as an approximation of the standard deviation of both old item distributions. These estimated starting parameters were used to sample ten sets of starting parameters from normal distributions. Another twenty sets of starting parameters were sampled from normal and uniform distributions with means, standard deviations and bounds that were not estimated from participant data. This was intended to provide a broad range of plausible starting parameters to avoid local minima in the model fitting procedure. According to the method described by Dunn (2010), the non-negative starting values were then log transformed, and all were input to the likelihood function which was optimized using the Nelder-Mead algorithm (as implemented in the optim function in R), giving 30 model fits per participant. The best of these model fits – that with the greatest log-likelihood – was chosen for each participant and used to give their parameter estimates.

The negative log-likelihood function for the UVSD model in Experiment 3 is

$$-\sum_{j=1}^{4} \sum_{i=1}^{6} N_{i,j}\times\log\left[ \Phi\left( I_{i+1}, \mu_{j},\sigma_{j} \right)- \Phi\left( I_{i},\mu_{j},\sigma_{j} \right) \right]$$

where *i* is the criterion index, *j* represents each condition (old-high, old-low, new-high, new-low; thus N_i,j_ is the number of responses per response category, per condition), Φ is the normal cumulative distribution function, and I = {-∞, C1, C2, … , CI, ∞} is a vector of criteria with upper and lower bounds of positive and negative infinity.
